# The gateway theory: bridging neural and immune interactions in the CNS

**DOI:** 10.3389/fnins.2013.00204

**Published:** 2013-10-29

**Authors:** Daisuke Kamimura, Moe Yamada, Masaya Harada, Lavannya Sabharwal, Jie Meng, Hidenori Bando, Hideki Ogura, Toru Atsumi, Yasunobu Arima, Masaaki Murakami

**Affiliations:** JST-CREST, WPI Immunology Frontier Research Center, Graduate School of Frontier Biosciences, Graduate School of Medicine, Osaka UniversitySuita, Osaka, Japan

**Keywords:** inflammation, cytokines, autoimmune diseases, inflammatory diseases, multiple sclerosis, gateway theory, neural activation, blood-brain barrier

## Abstract

The central nervous system (CNS) is considered an immune-privileged tissue protected by a specific vessel structure, the blood-brain barrier (BBB). Upon infection or traumatic injury in the CNS, the BBB is breached, and various immune cells are recruited to the affected area. In the case of autoimmune diseases in the CNS like multiple sclerosis (MS), autoreactive T cells against some CNS-specific antigens can theoretically attack neurons throughout the CNS. The affected CNS regions in MS patients can be detected as multiple focal plaques in the cerebrum, thoracic cord, and other regions. Vision problems are often associated with the initial phase of MS, suggesting a disturbance in the optic nerves. These observations raise the possibility that there exist specific signals that direct autoreactive T cells past the BBB and into particular sites of the CNS. Using a mouse model of MS, experimental autoimmune encephalomyelitis (EAE), we recently defined the mechanism of the pathogenesis in which regional neural stimulations modulate the status of the blood vessel endothelium to allow the invasion of autoreactive T cells into specific sites of the CNS via the fifth lumbar cord. This gate for autoreactive T cells can be artificially manipulated by removing gravity forces on the hind legs or by electric pulses to the soleus muscles, quadriceps, and triceps of mice, resulting in an accumulation of autoreactive T cells in the intended regions via the activation of regional neurons. Gating blood vessels by regional neural stimulations, a phenomenon we call the gateway theory, has potential therapeutic value not only in preventing autoimmunity, but also in augmenting the effects of cancer immunotherapies.

## Introduction

The immune system is a sophisticated protective mechanism that coordinates many types of bone-marrow derived hematopoietic cells including T cells, B cells, dendritic cells, and macrophages among others that have evolved to combat infectious agents and cancerous cells in the body. Careful regulation is required to control the immune system, because uncontrollable immune reactions, or chronic inflammation, are harmful and considered the pathogenesis of many diseases. It has been long recognized yet not well elucidated that neural activation directly influences the immune system. For example, mentally ill stresses in mice promote tumor progression, while good stresses, or eustress, which are induced by an enriched housing environment with increased space, physical activity, and social interactions, suppress it (Cao et al., 2010; Feng et al., 2012; Hassan et al., 2013). In human patients with autoimmune diseases, it has been suggested that psychological stresses including depression and anxiety are associated with disease progression and relapse (Mitsonis et al., 2009; Srivastava and Boyer, 2010). Whereas systemic modulation of the immune system by the nervous system through the secretion of corticosteroid is relatively well characterized, the regional interplay between these two biological systems is just beginning to be elucidated. Here we discuss the regional neuro-immune interactions during chronic inflammations including our recent findings.

## Inflammation underlies many types of chronic diseases

Traditionally, inflammation has been defined as pathophysiological symptoms with dolor, calor, rubor, and tumor (Latin for pain, heat, redness, and swelling, respectively). We now know that these symptoms are the consequences of the local accumulation and activation of immune cells that produce soluble factors such as cytokines and lipid mediators. Therefore, it is not an oversimplification to define inflammation as a biological mechanism that locally recruits various immune cells followed by the disruption of local homeostasis. Inflammation, or the accumulation of immune cells, basically acts as a protective mechanism against infectious agents including viruses and bacteria. In fact, mice genetically lacking T lymphocytes, which secrete various cytokines to orchestrate the immune reactions (CD4+ helper T cells) and exert cytotoxic activities that kill unnecessary or undesirable cells, such as infected cells as well as cancer cells (CD8+ killer T cells), are unable to control pathogen invasion via inflammation induction. At the same time, inflammation has to resolve immediately after these incidences come to an end, since chronically persistent inflammation has a rather adverse effect. Autoimmune diseases such as rheumatoid arthritis (RA) and multiple sclerosis (MS) are good examples of diseases that involve chronic inflammation. Signs of inflammation (i.e., local accumulation of immune cells followed by dysregulation of local homeostasis) are evident in the joints and the central nervous system (CNS) of RA and MS patients, respectively. We have also found the similar phenomena in the target organs of animal models for these diseases. In addition, accumulating evidence indicates that chronic inflammation underlies the pathogenesis of many other types of disorders including neurodegenerative diseases, metabolic syndromes, and even psychiatric diseases (Hamdani et al., 2012; Tabas and Glass, 2013). Thus, understanding the molecular mechanisms that convert protective acute inflammation into harmful chronic inflammation and how chronic inflammation persists is essential to the development of novel therapeutic interventions for many diseases and disorders.

## The inflammation amplifier provides a molecular mechanism for chronic inflammation

### Establishment of a rheumatoid arthritis model, f759 mice

Interleukin (IL)-6 is a well-studied cytokine that promotes inflammation. In fact, IL-6 is found upregulated in the serum and affected areas of human diseases and disorders such as synovial fluids in the case of RA, while genome-wide association studies (GWAS) have revealed that the IL-6 gene is genetically associated with a wide range of diseases including autoimmunity, neurodegenerative diseases, and cancer (http://geneticassociationdb.nih.gov/). Therefore, the establishment of an IL-6-dependent disease model has been desired for understanding the pathogenesis of various inflammatory diseases. IL-6 signals emanating from IL-6 receptor complexes, gp130 and IL-6R, involve the activation of STAT3, the central transcription factor for the many biological effects of IL-6. SOCS3 is a target gene of IL-6 and known to negatively regulate the signal transduction of IL-6 by binding to the receptor subunit gp130 and Jak kinases (Kamimura et al., 2003; Murakami et al., 2004). Given that IL-6 is abundant in patients with inflammatory disorders, we hypothesized that persistent activation of IL-6/gp130 signaling might induce inflammatory diseases. We therefore generated a knock-in mouse with a point mutation at the binding site of SOCS3 in gp130, thereby enhancing the IL-6 signal pathway (Ohtani et al., 2000). As expected, this mouse strain, called F759 mice, develops RA-like joint diseases spontaneously (Atsumi et al., 2002).

### Discovery of the inflammation amplifier

**Figure 1:**
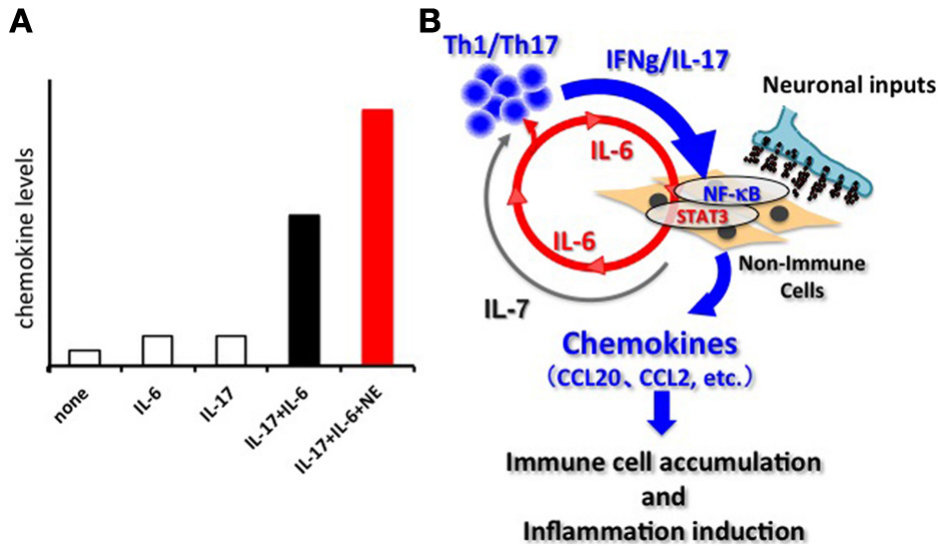
**The inflammation amplifier. (A)** Image of the activation of the inflammation amplifier in the presence and absence of NE. A combination of IL-17 and IL-6 causes a synergistic effect on the induction of inflammation-amplifier target chemokines such as CCL20 in non-immune cells including fibroblasts, endothelial cells, and certain synovial cells. NE further enhances the chemokine production. Other neurotransmitters can also influence activation of the inflammation amplifier. See Arima et al. (2012) for the original data. **(B)** The synergistic effect seen in **(A)** requires the concomitant activation of two transcriptions factors, NF-kB and STAT3. In the case of autoimmune diseases, Th-17 derived IL-17 induces an initially low amount of IL-6 from non-immune cells via NF-κ B activation. Secreted IL-6 together with IL-17 forms a positive feedback loop to produce excessive expression of the target genes including many inflammatory chemokines such as CCL20, CCL2, etc. Consequently, various immune cells are recruited at the region around the target cells via chemokine expression, and inflammation takes place. Persistent activation of the inflammation amplifier, as is the case in F759 mice, drives chronic inflammation. IL-7, a target of the amplifier and derived from non-immune cells, fuels the proliferation, and survival of Th17 cells. The activation of the inflammation amplifier can be modulated by regional neural activation.

Breeding experiments with various deficient mouse strains showed the joint disease in F759 mice mainly relies on CD4+ T cells, IL-6, and IL-17, which is a recently reported helper T-cell-derived cytokine involved in autoimmunity and activation of the transcription factor NF-κB (Sawa et al., 2006; Ogura et al., 2008). Indeed, the number of an IL-17-secreting CD4+ T cell subtype (Th17 cells) and the serum levels of IL-17 were increased in F759 mice. Furthermore, systemic expressions of IL-17 or IL-6 in F759 mice accelerated the disease onset and, interestingly, induced the production of IL-6 itself, suggesting a positive feedback loop (Ogura et al., 2008). Moreover, IL-17 expression in F759 mice induced an excessive level of IL-6 and chemokines. *In vitro* experiments using fibroblasts confirmed that IL-6 is a target of the simultaneous treatment of IL-17 and IL-6, but not of either cytokine alone (Ogura et al., 2008). DNA microarray analysis revealed that many chemokines are also targets of this synergism. Since chemokines recruit immune cells to promote inflammation via the dysregulation of local homeostasis, we named this synergistic mechanism the inflammation amplifier (formerly, IL-6 amplifier) (Figure 1) (Murakami and Hirano, 2012). Activation of the inflammation amplifier in endothelial cells is induced by the simultaneous activation of NF-κB and STAT3 followed by chemokine expressions. We expected that NF-κB and STAT3 stimulants, whether they are pro-inflammatory or anti-inflammatory cytokines, should enhance the expression of the chemokines. However, anti-inflammatory cytokines might indirectly suppress the activation of the inflammation amplifier by inhibiting activated helper T cells, which express various NF-κB and STAT3 stimulants including IL-17, IL-6, and TNFα. Moreover, as discussed in detail below, the inflammation amplifier, which can be regulated by neural inputs such as norepinephrine (NE), enhances the production of chemokines via NF-κB activation (Figure 1) (Arima et al., 2012).

### The inflammation amplifier is associated with various human diseases and disorders

Mice deficient in gp130 or STAT3 in fibroblasts and/or endothelial cells (i.e., inflammation amplifier-defective non-immune cells) are highly resistant to animal disease models including RA, MS, and allogeneic graft rejection, demonstrating that the inflammation amplifier is critical for multiple chronic inflammatory diseases *in vivo* (Ogura et al., 2008; Murakami et al., 2011; Arima et al., 2012; Lee et al., 2012). To explore the molecular mechanisms underlying the inflammation amplifier, we recently conducted genome-wide functional screening and identified around 1700 candidates that positively regulate it.

Interestingly, genes associated with human diseases including not only autoimmunity, but also metabolic syndromes, neurodegenerative diseases, and other inflammatory diseases including allergies and atopic dermatitis were significantly enriched in the candidate genes (Murakami et al., 2013). Based on these findings, we propose that de novo mutations, expression alterations, or epigenetic changes of these genes in non-immune cells during acute inflammation can cause dysregulation of the amplifier like that observed in F759 mice (i.e., a point-mutation of gp130), turning acute inflammation into a pathogenic chronic version.

## Neuro-immune interactions mediate immune homeostasis and pathogenesis

### Autoreactive CD4+ T cells enter the CNS from the fifth lumbar cord

MS is a chronic inflammatory disease in the CNS that, according to a recent genome-wide association study, involves CD4+ T cells (International Multiple Sclerosis Genetics et al., 2011). An animal model of MS, experimental autoimmune encephalomyelitis (EAE), is widely used to study the pathogenesis of MS, and autoreactive CD4+ T cells, in particular Th17 cells, have been demonstrated to be essential for the disease induction (Komiyama et al., 2006). In these experiments, mice or rats were immunized with CNS antigens such as myelin oligodendrocyte glycoprotein (MOG) or a proteolipid protein, resulting in the generation of autoreactive CD4+ T cells that target the CNS and the development of MS-like symptoms including progressive paralysis, particularly in the lower body. EAE can also be induced by a passive transfer method that is performed via the intravenous transfer of these autoreactive CD4+ T cells into naïve recipient animals. Indeed, the passive transfer method is suitable to track the behavior of the disease-causing CD4+ T cells without affecting the immune system systemically by adjuvant treatment. We have shown using the transfer method-induced EAE that the inflammation amplifier is critically involved in EAE development, because the disease symptoms were significantly ameliorated in amplifier-deficient mice such as type-1 collagen cre/gp130flox mice (Ogura et al., 2008; Arima et al., 2012). These results challenge traditional theories, because the blood-brain barrier (BBB) should block the migration of the pathogenic CD4+ T cells into the CNS. We thus sought the initial site where autoreactive CD4+ T cells invade the CNS during the development of EAE. A clinical symptom of typical EAE constantly begins with a loss of tonicity in the tail tip, suggesting immune attacks at a particular site in the lower body despite the uniform presence of the autoantigens in the CNS. Whole-mount sections of adult mice at a preclinical stage of EAE revealed that MOG-reactive CD4+ T cells preferentially entered the CNS from the dorsal vessels of the fifth lumbar (L5) cord (Arima et al., 2012).

### Dorsal vessels of the L5 cord express various chemokines via activation of the inflammation amplifier, even at steady state

This accumulation of CD4+ T cells is dependent on the inflammation amplifier, as expected. In addition, the treatment of mice with antibodies against CCL20, a chemokine potently recruiting Th17 and a target gene of the inflammation amplifier, suppressed the CD4+ T cell accumulation at the L5 dorsal vessels, suggesting that amplifier-mediated CCL20 overproduction causes the recruitment of pathogenic Th17 at the L5 cord (Arima et al., 2012). In fact, not only CCL20, but also various chemokines were upregulated at L5 dorsal vessels during EAE as compared to dorsal vessels from the other spinal levels. Unexpectedly, the upregulation of chemokines at L5 vessels was observed even under steady state (i.e., the natural condition). Given that a certain number of immune cells are present in the CNS, probably for the purpose of immune surveillance, and that the CNS-resident microglia is of bone-marrow origin, the increase of chemokines at the L5 cord may function as a gate for their precursors from the periphery to the CNS, although further studies are needed to confirm this conclusion.

### Regional neural activation induced by gravity plays a role in chemokine expression at the L5 gate

**Figure 2:**
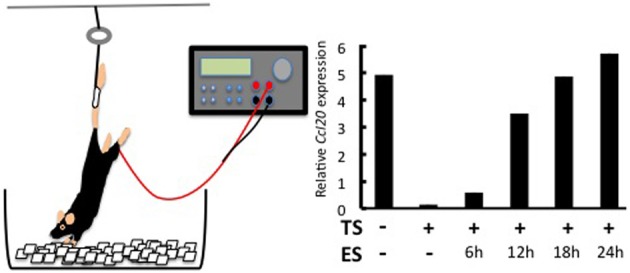
**Effects of regional neural activation on the invasion of Th17 cells into the CNS.** Tail suspension and electric stimulation of the soleus muscles **(left)**. Tail suspension (TS) reduces CCL20 expression in the L5 dorsal vessel. Electric stimulations (ES) to the soleus muscles of TS mice restore the chemokine expression in a time-dependent manner **(right)**. See Arima et al. (2012) for the original data.

Why the L5 is the location of the gate was answered by investigating a neuro-immune interaction. It is known that sensory neurons in the dorsal root ganglion (DRG) beside the L5 cord are connected to the soleus muscles, the main anti-gravity muscles (Ohira et al., 2004). We considered whether constant stimulation of the soleus muscles by a gravity force might mediate entry of the MOG-specific Th17 cells at the L5 cord. Indeed, when mice were tail-suspended so that the hind limbs were released from the gravity stimuli (Figure 2), MOG-specific Th17 cells no longer accumulated at the L5 cord. Instead, these Th17 cells accumulated at cervical cords as if a new gate was opened by a gravitational burden on the fore limb muscles (Arima et al., 2012). Consistently, the tail suspension suppressed CCL20 mRNA expression in the L5 dorsal blood vessels and decreased the expression of the neural activation marker c-fos in the L5 DRG. Moreover, artificial electric stimulations to the soleus muscles of the tail-suspended mice restored CCL20 expression (Figure 2) and Th17 accumulation at the L5 cord, as well as c-fos levels in the L5 DRG (Arima et al., 2012). These results strongly suggest that neural inputs from the soleus muscles in response to the gravity force play a role in activating the inflammation amplifier and lead to the expression of various chemokines including Th17-attracting CCL20 in L5 dorsal blood vessels.

### A sensory-sympathetic interaction is critical for the L5 entry of the autoreactive T cells

**Figure 3:**
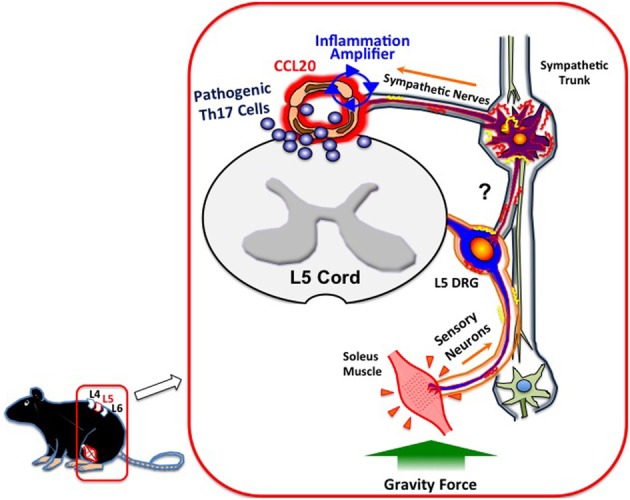
**Gateway for autoreactive T cells into the CNS.** Continuous stimulation of the soleus muscles by gravity leads to the constant activation of L5 DRG sensory neurons, followed by the activation of sympathetic neurons at the L5 region via unidentified circuits (?). NE, a neurotransmitter from sympathetic neurons, augments the inflammation amplifier at the dorsal vessels of L5, causing an elevation of CCL20 and subsequent Th17 infiltration into the CNS.

What mechanisms do afferent sensory neurons from the soleus muscles use to regulate the status of blood vessels at L5? Although a precise neural network remains elusive, we have shown sympathetic nerves to be involved. Blood flow speeds at L5 dorsal vessels become slower when mice are tail suspended, while electronic stimulation of the soleus muscles increases the flow speed. These results clearly suggest that autonomic nerves including sympathetic neurons are involved in the response to the tail suspension. In addition, blood flow speeds in blood vessels other than the L5 region, such as the femoral vessels, brain surface vessels, and the portal vein, are not affected by tail suspension (Arima et al., 2012). Furthermore, treatment with atenolol, a β 1 adrenergic receptor antagonist, or prazosin, an α1 adrenergic receptor antagonist, significantly inhibits CCL20 mRNA expression, NF-κB mRNA expression, and MOG-reactive Th17 accumulation at L5 vessels and also suppresses clinical signs of EAE (Arima et al., 2012). Consistent with these *in vivo* results, the addition of NE, which is a neurotransmitter from sympathetic neurons, to a culture of endothelial cells enhances chemokine expressions mediated by the activation of the inflammation amplifier (Figure 1A). Thus, anti-gravity responses of the soleus muscles lead to sympathetic nerve stimulation, creating a gateway for immune cells to pass through the CNS via L5 dorsal vessels (Arima et al., 2012). Based on these findings, we concluded that MOG-reactive, disease-causing Th17 cells make use of the L5 gateway to infiltrate the CNS and induce local inflammation by producing cytokines like IL-17 and IL-6, which further induces chemokines via the inflammation amplifier in parenchymal non-immune cells and results in chronic inflammation in the CNS (Figure 3). Thus, the neural response triggered by the gravity force creates a gate for immune cells to invade the CNS, although we also hypothesize that the clearance and/or apoptosis of immune cells in the CNS may contribute to the feed-forward mechanism of continued neuroinflammation (Goldmann et al., 2006). Our findings also imply that various types of neural activity affect the disease status differently. Indeed, we found and are currently studying the mechanism for how certain physical or mental stresses cause the deterioration or relapse of EAE in mice, which will shed new light on the molecular mechanism of the relapse and remission of MS.

## The gateway theory

**Figure 4:**
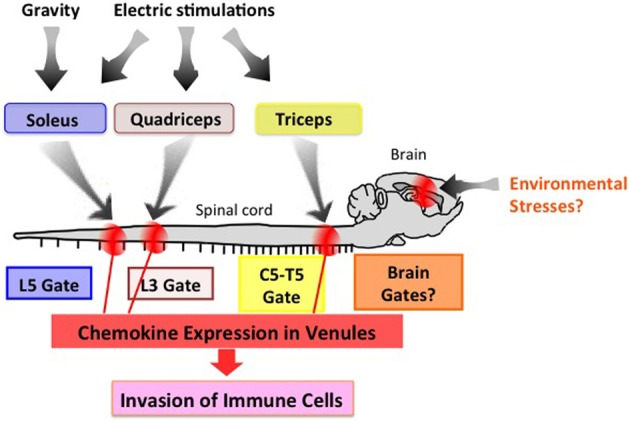
**The gateway theory.** Electric pulses to the upper arm, thigh, and soleus muscles induce activation of the inflammation amplifier (chemokine upregulation), thereby opening a gate in the dorsal vessels of the fifth cervical to fifth thoracic (C5-T5), third lumbar (L3), and fifth lumbar (L5) cords, respectively. Environmental stresses such as those from social interactions and mental stresses might create a specific gate in the brain. This effect is potentially an attractive target for manipulating immune cell migration *in vivo*.

Neuronal innervation occurs throughout the body and appropriately controls all organs. One potential strategy to therapeutically manage neuro-immune interactions is to find and stimulate the appropriate peripheral neurons connected to the organs to be treated. To test this theory, we selectively stimulated sensory neurons via electric stimulations of different muscles and observed local chemokine expressions in the vessels of spinal cords as a marker of the inflammatory response. Thigh muscles including the quadriceps are known to be regulated by L3 DRG neurons. Interestingly, electronic stimulation of these muscles followed by L3 DRG activation led to an increased expression of CCL20 in the L3 cord dorsal vessels of mice (Arima et al., 2012). In a similar fashion, chemokine levels in the fifth cervical to fifth thoracic cord vessels were upregulated by stimulations of the epitrochlearis/triceps brachii and upper arm muscles, are controlled by neurons located at the corresponding areas (Figure 4) (Arima et al., 2012). Based on these results, we propose “the gateway theory,” which describes blood vessels that act as gates for immune cell infiltration into organs and is dependent on regional neural stimulations. The theory is supported by empirical evidence that shows stresses from environments can affect the disease status and trigger relapse in MS patients. Experimentally, we have found that certain environmental stresses influence the disease progression in EAE mice by creating gates for autoreactive helper T cells to enter the CNS (manuscript in preparation). We hypothesize that these stresses create gates at certain CNS vessels via neural control of the inflammation amplifier to modify the disease status at specific vessels (Figure 4).

## Future directions

Investigations on whether the gateway theory can be more generally applied to tissues beyond the CNS are currently ongoing. If true, artificial manipulation of the gate should have significant clinical benefits, as closing it in normal cells would dampen autoimmune inflammation in a target organ without any systemic immune suppression, while opening it in tumors could enhance cancer immunotherapy. The precise molecular mechanisms for gating still need to be studied, however, to confirm this possibility.

### Conflict of interest statement

The authors declare that the research was conducted in the absence of any commercial or financial relationships that could be construed as a potential conflict of interest.
